# Personalize, participate, predict, and prevent: 4Ps in inflammatory bowel disease

**DOI:** 10.3389/fmed.2023.1031998

**Published:** 2023-04-11

**Authors:** Marco Vincenzo Lenti, Maria Lia Scribano, Livia Biancone, Rachele Ciccocioppo, Daniela Pugliese, Luca Pastorelli, Gionata Fiorino, Edoardo Savarino, Flavio Andrea Caprioli, Sandro Ardizzone, Massimo Claudio Fantini, Gian Eugenio Tontini, Ambrogio Orlando, Gianluca Matteo Sampietro, Giacomo Carlo Sturniolo, Giovanni Monteleone, Maurizio Vecchi, Anna Kohn, Marco Daperno, Renata D’Incà, Gino Roberto Corazza, Antonio Di Sabatino

**Affiliations:** ^1^Department of Internal Medicine and Medical Therapeutics, University of Pavia, Pavia, Italy; ^2^Department of Internal Medicine, San Matteo Hospital Foundation, Pavia, Italy; ^3^Villa Stuart, Multi-Speciality Clinic, Rome, Italy; ^4^Unit of Gastroenterology, Department of Systems Medicine, University of Rome "Tor Vergata", Rome, Italy; ^5^Gastroenterology Unit, Department of Medicine, A.O.U.I. Policlinico G.B. Rossi and University of Verona, Verona, Italy; ^6^CEMAD Digestive Disease Center, Fondazione Policlinico Universitario "A. Gemelli" IRCCS, Università Cattolica del Sacro Cuore, Rome, Italy; ^7^Liver and Gastroenterology Unit, ASST Santi Paolo e Carlo, Milan, Italy; ^8^Department of Health Sciences, University of Milan, Milan, Italy; ^9^IBD Unit, Ospedale San Camillo-Forlanini, Rome, Italy; ^10^Department of Gastroenterology, San Raffaele Hospital and Vita-Salute San Raffaele University,, Milan, Italy; ^11^Gastroenterology Unit, Department of Surgery, Oncology and Gastroenterology, University of Padua, Padua, Italy; ^12^Gastroenterology and Endoscopy Unit, Fondazione Istituto di Ricovero e Cura a Carattere Scientifico Cà Granda, Ospedale Maggiore Policlinico and Università degli Studi di Milano, Milan, Italy; ^13^Gastroenterology and Digestive Endoscopy Unit, ASST Fatebenefratelli Sacco, Milan, Italy; ^14^Department of Medical Science and Public Health, University of Cagliari, Cagliari, Italy; ^15^Gastroenterology Unit, Azienda Ospedaliero-Universitaria (AOU) di Cagliari, Cagliari, Italy; ^16^Department of Pathophysiology and Transplantation, Fondazione IRCCS Ca' Granda Ospedale Maggiore Policlinico, University of Milan, Milano, Italy; ^17^Inflammatory Bowel Disease Unit, Azienda Ospedaliera Ospedali Riuniti "Villa Sofia-Cervello" Palermo, Palermo, Italy; ^18^Division of General and HBP Surgery, Rho Memorial Hospital, ASST Rhodense, Rho, Milano, Italy; ^19^Gastroenterology Operative Unit, Azienda Ospedaliera San Camillo-Forlanini FR, Rome, Italy; ^20^Division of Gastroenterology, Ospedale Ordine Mauriziano di Torino, Turin, Italy

**Keywords:** clinical complexity, Crohn’s disease, internal medicine, precision medicine, ulcerative colitis

## Abstract

Inflammatory bowel disease (IBD), which includes Crohn’s disease (CD) and ulcerative colitis (UC), is a complex, immune-mediated, disorder which leads to several gastrointestinal and systemic manifestations determining a poor quality of life, disability, and other negative health outcomes. Our knowledge of this condition has greatly improved over the last few decades, and a comprehensive management should take into account both biological (i.e., disease-related, patient-related) and non-biological (i.e., socioeconomic, cultural, environmental, behavioral) factors which contribute to the disease phenotype. From this point of view, the so called 4P medicine framework, including personalization, prediction, prevention, and participation could be useful for tailoring *ad hoc* interventions in IBD patients. In this review, we discuss the cutting-edge issues regarding personalization in special settings (i.e., pregnancy, oncology, infectious diseases), patient participation (i.e., how to communicate, disability, tackling stigma and resilience, quality of care), disease prediction (i.e., faecal markers, response to treatments), and prevention (i.e., dysplasia through endoscopy, infections through vaccinations, and post-surgical recurrence). Finally, we provide an outlook discussing the unmet needs for implementing this conceptual framework in clinical practice.

## Introduction

**Figure 1:**
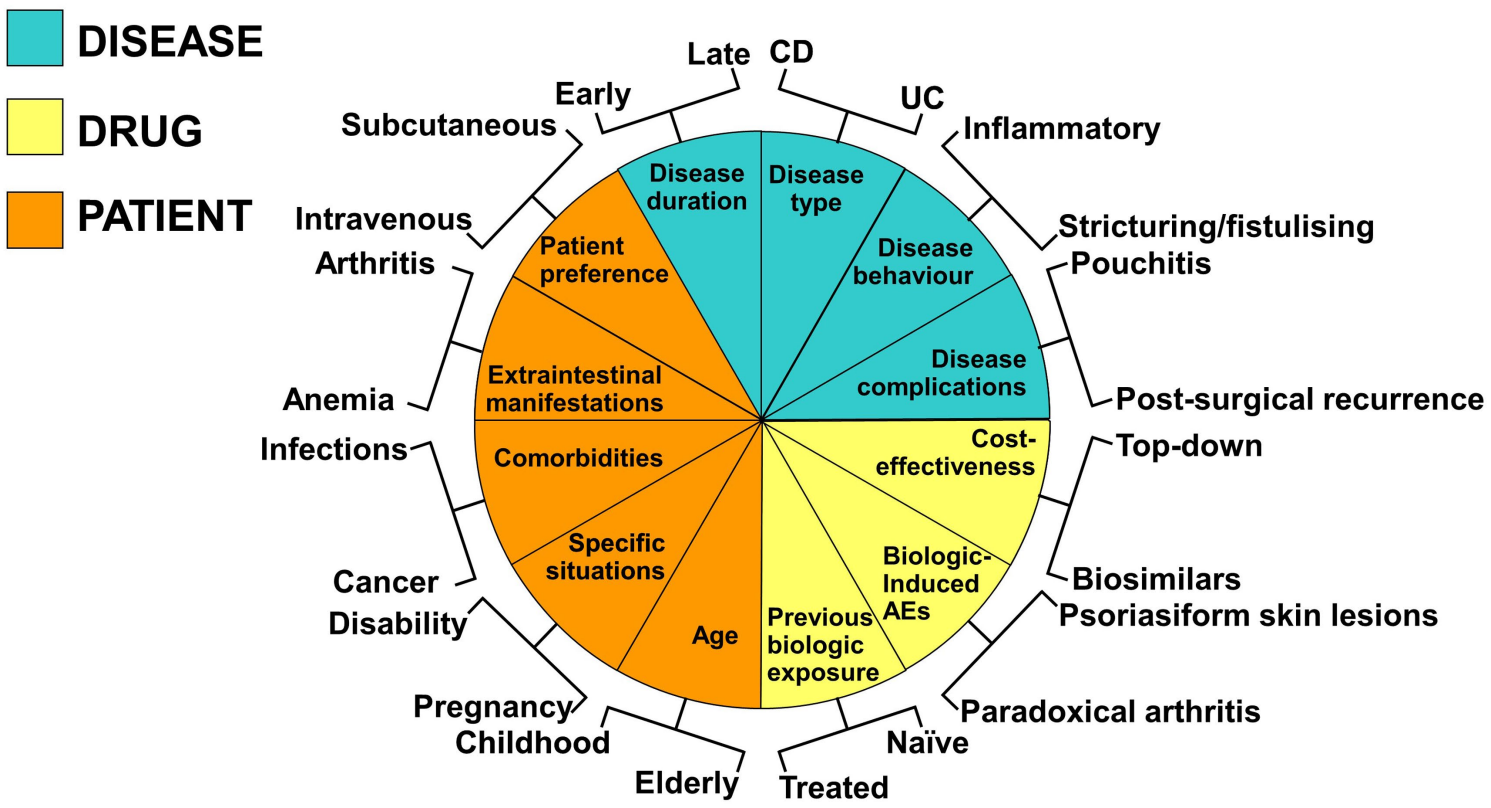
Schematic representation of disease-, drug-, and patient-related factors to be considered in the overall management of inflammatory bowel disease (IBD). The factors depicted in the Figure encompasses the four main domains of the 4P medicine, including prediction, prevention, participation, and personalization. Current guidelines do not comprehensively cover these aspects, that all pertain to an internal medicine setting. Indeed, many other variables not depicted in the Figure should also be considered and discussed with patients.

Inflammatory bowel disease (IBD) is an immune-mediated, lifelong, chronic, and disabling condition (1–4), which is constantly increasing worldwide (5), affecting individuals of any age and gender. IBD includes two main entities, namely ulcerative colitis (UC) and Crohn’s disease (CD), both causing intestinal (e.g., abdominal pain, diarrhoea and/or constipation, rectal bleeding, perianal symptoms) and extraintestinal (e.g., fatigue, anaemia, micronutrient deficiencies, arthritis, fertility issues) manifestations, deeply affecting patients’ quality of life, and social and sexual relationships (1, 2, 4). Over the last three decades, our knowledge about this condition has greatly improved, and the treatment paradigm has shifted from the use of unselective anti-inflammatory and immunosuppressive agents (e.g., mesalamine, corticosteroids, methotrexate, azathioprine, 6-mercaptopurine) to the use of more selective, pathogenesis-oriented, therapies (e.g., biological agents, small molecules) (6). Given all the proteiform and multifaceted aspects that should be taken into account, namely patient-, disease-, and drug-related factors (Figure 1), IBD can be considered as a clear example of a clinically complex disease (7), in which both biological and non-biological factors play a role in determining patients’ outcomes.

For these reasons, IBD should be considered as a systemic disorder, rather than as a specialty-oriented condition. In fact, although authoritative guidelines are available (8–11), they do not comprehensively consider all the possible aspects that may influence decision-making and patients’ outcomes, such as specific age groups (e.g., childhood, age of transition, elderly), specific situations (e.g., pregnancy and lactation, poor medication adherence, socioeconomic deprivation), and comorbidities (e.g., cancer, autoimmunity, infectious diseases). From this point of view, the so called 4P medicine (12), which stands for predictive, preventive, personalized, and participatory, might be considered as a proper framework for tailoring interventions for IBD at a patient—rather than at a disease—level, depending on their specific needs. Three main drivers have been implicated in the emergence of the 4P medicine, including the expansion of systems biology and systems medicine, the digital revolution, and the consumer-driver healthcare and social networks (12). All of these have indeed fuelled much interest in more precise and personalized medicine.

In this narrative review, within the framework of the 4P medicine, we have chosen a series of the most controversial and the currently debated issues in IBD, with an expert-based approach. Specifically, we have chosen three main topics per each “P” included in the framework. Additionally, we have also commented on the unmet needs in the field of IBD, which could soon be implemented.

No new data were generated or analyzed in support of this paper.

## Personalize

### Tailoring treatment of IBD in pregnant women

IBD commonly affects most patients in their reproductive age. Women with IBD experience significant concerns when considering pregnancy, especially related to the potential risks of drugs on pregnancy and foetal development, which may lead to a poor adherence to the therapy (13). It is of paramount importance to achieve a stable disease remission prior to conception and maintain a quiescent disease throughout pregnancy (14). Therefore, patients must be aware that if the treatment is discontinued, the risk of maternal and foetal complications related to disease relapse outweighs the potential adverse effects of medications. Preconception counseling is recommended, as it can improve drug adherence and pregnancy outcomes (15).

Many studies have evaluated the impact of therapy on the course and outcome of pregnancy in IBD women. However, determining the safety of drugs in this context is challenging as several factors such as disease activity, concomitant therapies, comorbidities and other maternal aspects can confound the study results. Most drugs have a favorable safety profile for use in pregnant patients (Table 1). Aminosalicylates are considered safe, therefore guidelines recommend their continuation throughout pregnancy (16, 17). In women who have an IBD flare during pregnancy, corticosteroids may be employed (16–18). The PIANO registry, including 1,490 pregnant women with IBD, showed an association between corticosteroid use and preterm birth, intrauterine growth restriction and low birthweight (19). However, the risk might be associated with IBD activity rather than drug use. Budesonide may be preferred for the treatment of mild-to-moderate CD relapse (17). The antibiotics metronidazole and ciprofloxacin should be avoided during pregnancy, especially in the first trimester (16). An increased rate of preterm birth has been observed with use of thiopurines (4–6). However, more recent data reported no heightened risk of adverse pregnancy outcomes associated with these medications (20, 21). Therefore, therapy with thiopurines should be maintained throughout pregnancy because the benefits outweigh potential risks (17). Methotrexate is absolutely contraindicated in pregnancy, due to its teratogenic and abortifacient action (16–18). Evidence on the use of cyclosporine in IBD patients is limited to pregnant women with a severe UC refractory to treatment (22). An increased rate of prematurity and low birthweight has been observed.

**Table 1:** Medical management of IBD during fertility and pregnancy.

Medication	Pregnancy safety	Recommendations
Aminosalicylates		
Mesalazine	Low risk	All mesalazine formulations are now phthalate-free. Dibutyl phthalate coating reported to be teratogenic in animals
Sulfasalazine	Low risk	Folate supplementation: 2 mg/day
Corticosteroids		
Systemic corticosteroids	Low risk	Use for IBD relapse. Possible increased risk of cleft lip/palate (first trimester exposure), not confirmed by all studies
Budesonide	Low risk, limited data	Use for ileocaecal CD relapse
Antibiotics		
Metronidazole	Avoid in the first trimester	Short-term courses in second and third trimesters for pouchitis and perianal CD Possible increased risk of cleft lip/palate (first trimester exposure)
Ciprofloxacin	Avoid in the first trimester	Short-term courses in second and third trimesters for pouchitis and perianal CD Potential toxicity to cartilage (first trimester exposure)
Immunomodulators		
Thiopurines	Low risk	When combined with biologic agents consider stopping in appropriate women, given a possible increased risk of infant infections
Methotrexate	Contraindicated	Must be discontinued 3–6 months before attempting conception
Cyclosporine	Unclear, limited data	Rescue therapy in refractory UC to avoid colectomy
Biologics Anti-TNF		
Infliximab	Low risk	Consider discontinuing the drug around gestational week 24–26
Adalimumab	Low risk	Consider discontinuing the drug around gestational week 24–26
Golimumab	Low risk	Consider discontinuing the drug around gestational week 24–26
Certolizumab pegol	Low risk	Continue throughout pregnancy
Anti-α4β7 integrin		
Vedolizumab	Likely low risk, limited data	Individualized decision
Anti-interleukin 12–23		
Ustekinumab	Likely low risk, limited data	Individualized decision
Janus kinase inhibitor		
Tofacitinib	Contraindicated, limited data	The manufacturer recommendation is to use contraception during treatment with the drug

IBD, inflammatory bowel disease; CD, Crohn’s disease; UC, ulcerative colitis; TNF, tumor necrosis factor.

Results of several studies suggest low risk for use of anti-tumor necrosis factor (TNF) agents during pregnancy (23). In the PIANO and TREAT registers no increase in congenital abnormalities or adverse pregnancy outcomes was found among women who received anti-TNFs compared to the unexposed IBD control group (21, 24). The anti-TNF drugs, aside from certolizumab pegol, cross the placenta in the second and, especially, in the third trimester. Discontinuing the therapy around gestational week 24–26 is recommended by European guidelines to reduce anti-TNF exposure of the foetus (16). Indeed, this is recommended for patients who are in stable and complete remission, otherwise this therapy can be continued. Some studies, including the PIANO registry and the European TEDDY study, showed that anti-TNF use throughout pregnancy was not associated with an increased rate of infantile infections or serious infections (21, 25). Stopping anti-TNF therapy before the third trimester is an individualized decision based on both IBD activity and the woman’s risk profile (18).

Available data on the use of vedolizumab in pregnancy is limited. A retrospective study of 24 pregnancies exposed to vedolizumab reported some maternal and neonatal complications (26). Different results arise from a prospective comparison study on 24 pregnancies in 21 women treated with vedolizumab, whose use appeared low risk (27). Likewise, no concerns were observed in the case–control European CONCEIVE study, or in the PREGNANCY-GETAID study (28, 29). Therefore, the use of vedolizumab during pregnancy seems to convey a low risk, however no firm conclusion can be drawn, and a personalized decision should be made.

The effects of ustekinumab in pregnant women with IBD were evaluated in 29 pregnancies in the PREGNACY-GETAID study, without negative signals on maternal or neonatal outcomes (30). This data was confirmed by the preliminary results from the DUMBO prospective registry (31). Thus, ustekinumab appears to be safe during pregnancy, however the experience is limited, and its use should be individualized.

Tofacitinib, at doses exceeding human dose, is teratogenic in animals (18). Limited reported outcomes of pregnancy and new-borns in women on tofacitinib appeared to be similar to those observed in the general population (31, 32). Nevertheless, at present, the use of tofacitinib during pregnancy is contraindicated.

### Tailoring treatment of IBD in cancer patients

Cancer risk is increased in IBD patients with a history of cancer, regardless of IBD-related treatments, as in the general population. The risk of new or recurrent cancer in patients with a present or past history of cancer using immunomodulators is under investigation. Most of these studies include IBD patients with less invasive cancer types, associated with a low risk of recurrence and limited follow up, thus limiting this analysis. The frequent combined conventional immunomodulators and biologics also does not allow an appropriate assessment of the risk of new/recurrent cancer using each specific treatment in IBD. Moreover, the time interval between diagnosis or remission of cancer and immunomodulators use differ among studies. Finally, patients with less severe cancer or with a lower risk of recurrence has mostly been considered. Overall, a joint management with oncologists shared with patients and follow up tailored on a patient’s basis is required when considering immunomodulators in IBD patients with a history of cancer.

Thiopurines use in IBD has been associated with a higher risk of skin cancers, particularly non melanoma skin cancers and lymphoma, further increased by combined TNFα-antagonists (33, 34). A slightly increased risk of cervical abnormalities has also been suggested (33, 34). The few available evidences assessing the risk of new/recurrent cancer under thiopurines in IBD patients with previous cancer are encouraging (35), including a large meta-analysis in patients with a history of malignancy treated or untreated with thiopurines or other immunomodulators (36).Nevertheless, a careful evaluation of IBD patients with a history of cancer is required before using thiopurines and drug discontinuation at diagnosis of cancer currently appears the initial option. Cancer type, time interval from diagnosis of cancer, age and IBD severity should be considered, with decision made on a patients’ basis shared with oncologists and patients.

Evidence regarding the risk of new/recurrent cancer in IBD patients with present/prior cancer using methotrexate monotherapy is lacking and further studies are required. Not conclusive, but reassuring data derive using methotrexate in rheumatoid arthritis (36, 37).

TNF-α is a cytokine provided of several activities, including necrotic activity on cancer cells “*in vitro*.” Several large independent studies support that TNF-inhibitors do not increase the overall cancer risk in IBD (33, 38–42). However, a slight excess risk of skin cancers, particularly melanoma, has been reported together with an increased risk of lymphoma particularly using combined thiopurines (43, 44). Whether TNFα-antagonists increase the risk of new/recurrent cancer in IBD patients with a diagnosis of cancer is being investigated. This analysis is currently limited by the small samples size of patients with specific cancer types. Additional limitations include differences among studies in terms of cancer stage, concomitant treatments, length of follow up and time interval from diagnosis of cancer to TNF-antagonists use. Despite these limitations, available preliminary evidence (mostly referring to overall cancer or melanoma) does not support that blocking TNFα increases the risk of new/recurrent cancer in IBD patients with prior cancer (36, 45, 46). As for other cancers, indication for TNF-antagonists in IBD patients with a history of cancer requires a careful selection of patients in a multidisciplinary approach with oncologists.

Vedolizumab (anti-α4β7 integrin) and ustekinumab (anti-IL-21/IL-23) have recently been used in IBD. Although a longer follow up is required, evidences suggest that their use does not increase the overall cancer risk in IBD (47, 48).In patients with a present or prior history of cancer, one retrospective study reported that vedolizumab does not increase the risk of new/recurrent cancer (49). Comparable findings were observed using vedolizumab or ustekinumab in IBD patients with prior malignancy (50). Additional data and a longer follow up is required for assessing the risk of new/recurrent cancer using these more immunomodulators, and particularly tofacitinib (pan-JAK inhibitor) (51) in IBD patients with a history of cancer.

### Tailoring treatment of IBD in patients with superimposed viral infections

The main culprits of superimposed viral infection in IBD are human cytomegalovirus (HCMV, a β-herpesvirus) and Epstein–Barr virus (EBV, a ɣ-herpesvirus) (52, 53) They display the unique ability of establishing a lifelong latency (54–56) and reactivating in cases of reduced host immunity giving rise to systemic or end-organ disease (57, 58). Latent infection is the condition where viral genome is integrated with the host genome, viral gene expression is limited, and no virus is produced. The shift from latency to replication largely depends on T-cell immune control and/or changes in the differentiation/activation state of cells harboring viral genome (57, 58). While isolated reactivation events occur uneventfully in the immunocompetent host, critical stress or immunosuppression may trigger the lytic phase of the viral life cycle (57, 59). In this case, the involvement of colonic mucosa in IBD patients (60, 61) makes challenging to distinguish between a relapse of the underlying disease and a superimposed viral colitis. Indeed, both the clinical picture and endoscopic features are similar and viral DNA in peripheral blood is detectable only in a minority of patients, while serology is unreliable. The measurement of mucosal viral load by quantitative real-time polymerase chain reaction allows not only to diagnose a superimposed viral colitis, but also to distinguish this condition from infection by applying the cut-off value of 1,000 copies/105 cells, as follows:

- viral colitis for values >103 copies/105 cells,
- reactivation of latent infection for values between 102 and 103 copies/105 cells,
- latent infection for values <102 copies/105 cells,
- systemic disease when detectable copies are found in peripheral blood together with viral colitis.

**Figure 2:**
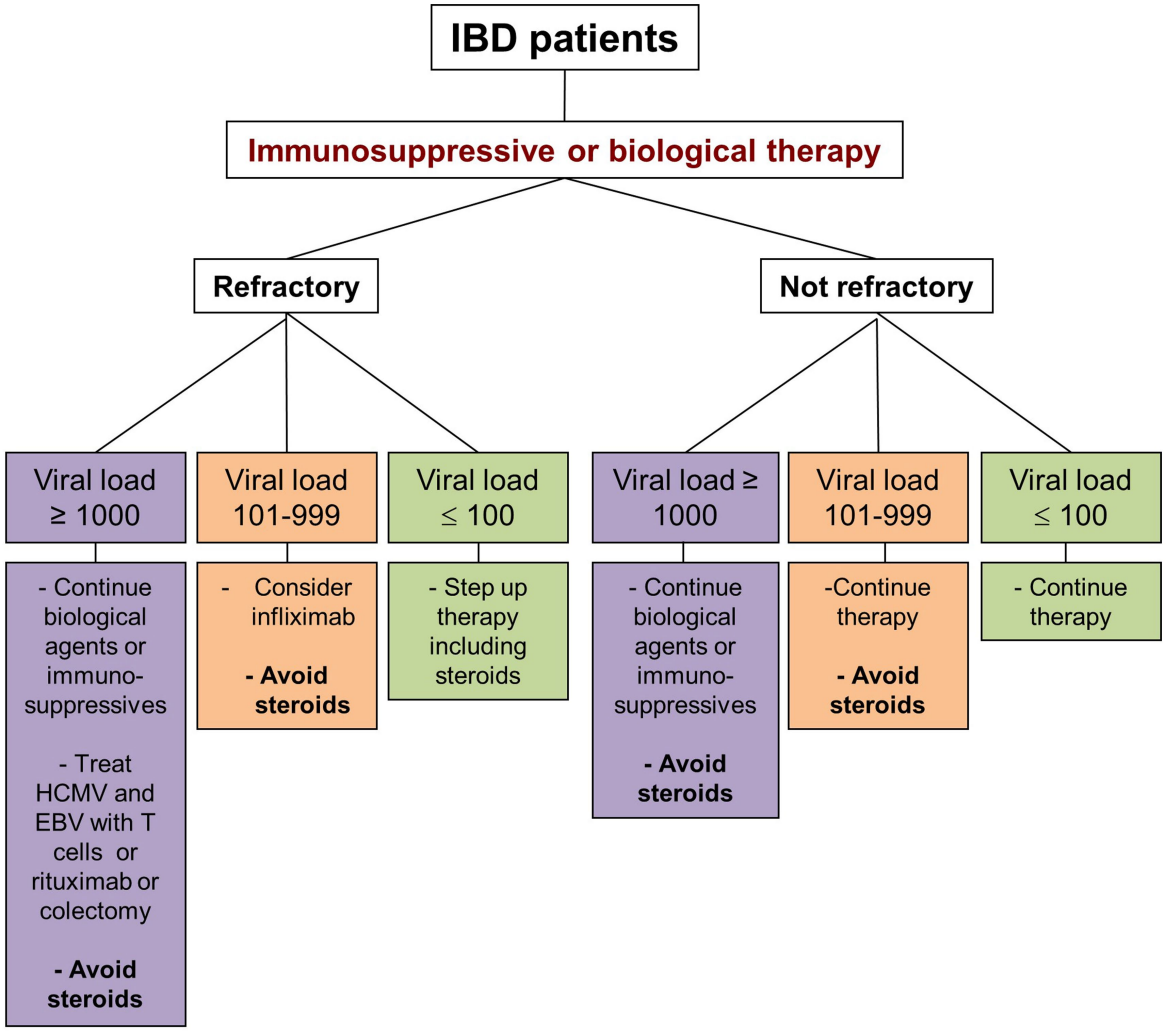
Schematic representation of the management of patients with inflammatory bowel disease treated with immunosuppressants or biologics and developing a viral colitis sustained by human cytomegalovirus (HCMV) and/or Epstein–Barr virus (EBV). Depending on colitis refractoriness and the viral load, different scenarios are depicted. The assessment of the viral load is crucial, as HCMV and EBV may be normally found in very low concentrations in the gut mucosa and are considered innocent bystanders in this case.

Indeed, both immunohistochemistry and *in situ* hybridization are poorly sensitive and even in case of positivity, they do not give any information about the state of virus replication (62). Moreover, a correct and timely therapeutic management depends on the stratification of patients accordingly to these value ranges (Figure 2) since in the case of viral colitis/systemic disease a quick tapering and discontinuation of steroid therapy is mandatory, since it favors viral replication (63), while it is only preferable in case of reactivation to avoid an overt viral colitis, and no modification is required in case of latent infection. As far as the underlying therapy with azathioprine or anti-tumor necrosis factor-α agents is regarded, their long-lasting therapeutic activity makes unnecessary their discontinuation, rather the ability of infliximab to reinduce T-cell apoptosis (64) may limit the activation of the viral lytic phase. By contrast, the effects of inhibitors of T-cell homing (such as anti-integrin monoclonal antibodies, modulators of sphingosine -phosphate receptor) are unpredictable since their use may hamper the number of effector memory T-cells that are largely responsible for the containment of virus-infected cells and virus replication (65). No data are available on the effects, if any, of small molecules (Janus kinase inhibitors) on EBV and HCMV reactivation in IBD patients. In addition, antiviral therapy with ganciclovir (5 mg/kg bid iv) for 4 weeks is recommended in those with HCMV disease (66), with monitoring of the mucosal viral load at the end of treatment, while no effective treatment is available for EBV disease. The use of the anti-CD20 monoclonal antibody rituximab (375 mg/m^2^ body surface iv weekly) (67) was tentatively applied, but the poorness of information does not allow to draw firm conclusion. The use of adoptive transfer of virus-specific cytotoxic T-cell clones (68) has great therapeutic potential, but it is limited to those centers with good manufacturing practice facility and approval by regulatory body is required. This strategy is even more important if considering that an impairment of EBV-specific T-cell immunity was shown in ulcerative colitis patients, mostly in those refractory to current therapy (69). Whether passive transfer of virus-specific antibodies or immunoglobulin preparations may help the immune system of IBD patients to recover the ability to silence viral replication deserves investigation. Finally, an increased risk of lymphoma has been found especially in young IBD males under thiopurines, where a role for primary EBV infection has been proposed (70). Therefore, it is mandatory to start this therapy only after positivity of EBV serology has been verified. In parallel, all those factors favoring viral reactivation, such as malnourishment, should be corrected.

## Participate

### How to communicate with IBD patients

An effective and adequate communication between patients and healthcare providers (HCP) constitutes a critical step in the management of IBD throughout the whole course of disease, indeed representing the foundation for a successful engagement in the decision-making process. As a matter of fact, playing an active role in the therapeutic strategies is ranked as “very important” by most of patients (71) and it is associated with a higher rate of satisfaction and adherence to therapy, as well as with improved clinical outcomes (72, 73). Education concerning the disease itself, its course, management, and prognosis starts at the time of diagnosis and requires a careful assessment of patients’ needs, concerns, and level of awareness by HCP (74). At this phase, several barriers could influence the quality of the communication, such as the diagnostic delay responsible for patients’ distrust in medicine and HCP, the difficult acceptance of a chronic disease and its potential impact on quality of life (75). A useful tool to assess patients’ concern and to plan specific interventions accordingly, is represented by the rating form of IBD patient concerns (RFIPC), a 25-item questionnaire, requiring an individual score for the level of concern about several topics (e.g., cancer, specific symptoms, drug safety) (76). A survey from University of Manitoba, enrolling newly diagnosed IBD patients, showed that about 25% of them were dissatisfied with the amount of information received at the time of diagnosis, especially on specific issues such as long-term prognosis, infertility risk and inheritance, therapeutic management of clinical symptoms and impact on daily living activities (77). Similar findings also emerged from the UC Narrative Global Surveys, including both 2,100 UC patients and 1,254 physicians, showing an overall satisfying doctor-patient relationship; nevertheless, it is worth pointing out that up to 72% of patients wished they had received more information and support at the time of the diagnosis. Moreover, specific themes, in particular those ones related to emotional domains, seem to represent a taboo for almost half of patients. Of note, sexual health and sexual dysfunction are among the most neglected topics during medical visits, for several generical barriers such as “lack of time” or “more urgent issue to discuss,” but also embarrassment perceived by both actors and the inadequate physicians’ competence on how to manage potential emerging problems (78, 79).

Among HCP, a key role for the establishment of an efficacious communication with patients is played by the IBD-nurses. In a study promoted by the Crohn’s and Colitis UK organization, 1,081 young adults IBD patients (mean age 23.3 years) were asked to respond to an online questionnaire about several issues related to their disease, including the quality of communication with HCP. Gastroenterologists were shown to be the main source of information, especially at the time of diagnosis. However, IBD-nurses usually represented the first contact during a flare, ranked highest for the frequency of contacts and for the mean level of comfort in communication, compared to gastroenterologists (*p* < 0.0001) (80).

Moreover, IBD-nurses often represent the gatekeeper and filter on lots of information derived from the patients’ online health information seeking (81).

A specific mention should be made in regard to the specific language adopted by physicians while communicating with patients about their health status and when addressing their specific concerns. In particular, the Food and Drug Administration presented some evidence-based practical advice for the communication of risk and benefits, including, among others: (1) avoiding excessive use of technical terms, which would increase the risk of misunderstanding and ineffective communication, (2) employing visual tools to ease the comprehension of particularly challenging issues, and (3) providing absolute risks – rather than relative risks – when discussing drug safety (82).

### Disability in IBD

In IBD patients, the altered bowel functions and non-bowel related symptoms (83, 84) heavily impact on patients’ work, social and home life activities, leading to various degrees of disability.

According to the World Health Organization (WHO) disability is defined as: “…any restriction or lack of ability to perform an activity in the manner or within the range considered normal for a human being” (85).

Recent data demonstrate that up to 46.6% of IBD patients complain some form of disability (86); given its relevance, researchers and clinicians felt the need to standardize the definition and the tools for assessing disability. The IBD-disability index was developed in 2011 and includes different aspects of life, namely General health, Body functions, Body structures, Activities and participation, Environmental factors (87). Although, this index appeared a solid and reliable research tool, it is not administered with ease in daily practice. As such, in 2017, the most important items from this questionnaire were selected and entered in a new visual instrument, called IBD-Disk, a self-administered tool (88).

Indeed, disease clinical activity in both CD and UC significantly correlates with disability, CD having a greater impact than UC (86, 89). Disability also strongly correlates to surgeries, hospitalizations, corticosteroid or anti-TNF usage. The presence of extraintestinal manifestations, as well as anaemia, has a profound impact on patients’ quality of life and disability as well (89). Patients with ileal pouch anal anastomosis had greater disability compared to UC patients with medically managed disease; female sex and having a public, rather than private, health insurance were further detrimental factors (90, 91). Also, overweight/obesity, being a social worker or having the perception of the need for a psychotherapist were identified as markers for a higher disability (86).

IBD-related disability also has consequences on working life and productivity (92). A Spanish community-based study revealed that 32% of patients had an officially recognized disability degree, while 4.1% had a work-disability pension; again, markers of a severe and progressive disease were major drivers of disability (93). Eventually, disability causes work loss and absenteeism, but also presenteeism (94) and affects patients’ perception of their working life (95). Indeed, work-related disability does have a detrimental impact on patients’ productivity, careers and earnings, which is specifically relevant for the female sex (96).

To conclude, preventing disability should be one of our pivotal aims when managing IBD patients. Disease control and avoiding disease progression may be a proper starting point; still, disability is multifaceted and not fully reflected by disease activity parameters. A new challenge for the years to come is to incorporate disability scores in clinical practice and trials (97).

### Stigmatization and resilience in IBD

Given the symptoms experienced by IBD patients and IBD-related treatments, it appears clear that stigmatization may constitute a major issue in this context (98–101). Stigmatization is defined as the identification of negative characteristics that distinguish a person as different and worthy of separation from their family, social, or working environments, leading to losing social status, discrimination, and isolation (102). The sigma “perceived” by patients over time may be “internalized,” thus leading to the belief that stereotypes about their condition are true and adapt their life to this new situation (4, 103). While stigmatization has been extensively investigated in patients living with chronic conditions having a high social impact, as in the case of mental illnesses (102), HIV infection (103), and cancer (104), only over the last decade has stigmatization drawn attention in the IBD field. Stigmatization can be quantified through different scales. Taft et al. (105) proposed the perceived stigma scale (PSS) which was, however, adapted from the original PSS in irritable bowel syndrome (106). The PSS-IBD is a self-administered scale comprising 10 statements which refer to either significant others or health care professionals. According to a recent review (4), roughly 30 papers have looked at stigmatization in IBD so far, highlighting some key areas of intervention, including the work/school environment, sexual life, social relationships, depression and anxiety. As stigmatization does not seem to depend on age, sex, and IBD clinical activity (98), other factors should be investigated for tackling this issue.

The concept of stigmatization may be related to that of resilience (4), which is the ability to adapt well in the face of adversity, tragedy, threats or significant sources of stress (107). Hence, resilience is the ability to cope positively and to “bounce back” despite an adverse event, and this characteristic has been studied in patients with different chronic illnesses (108–110), a higher resilience being generally correlated with better outcomes. Several scales have been proposed, the most used being the 25-item Connor-Davidson resilience scale (CD-RISC) (111), validated into more than 70 different languages. Despite the availability of effective interventions (112), resilience has been poorly addressed in IBD (4), and some results are contrasting. For example, in a recent Italian study (98), resilience was shown to be inversely related to disease activity in UC, but not in CD, while in a previous study from Korea (112), the clinical characteristics of UC did not affect resilience. Other factors that have been assessed in other studies are age, sex, disease characteristics, and social context, although with some limitations (4). The only study specifically looking at their mutual relationship found a significant—though modest—and inverse correlation between the two (98).

To conclude, stigmatization and resilience should be considered as important features in IBD patients. Future studies should investigate both their determinants and possible *ad-hoc* interventions for improving resilience and fighting stigmatization.

### Quality of care standards in IBD

IBD is a spectrum of inflammatory chronic intestinal conditions which need a complex approach in regards of diagnosis, management and follow-up (113). Despite several guidelines (8, 11, 114, 115), the approach to IBD patients remains heterogeneous across countries and even in the same country, as it depends on several factors, such as access to healthcare system, reimbursement policies, expertise of healthcare professionals and knowledge of IBD by the general population. Therefore, a clear definition of standards of care (SoC), and objective outcome measures in IBD are needed.

The most recent version of a general definition of SoC “that which a minimally competent physician in the same field would do under similar circumstances” (116). Based on this definition, the European Crohn’s Colitis Organisation (ECCO) decided to approach and define quality SoC by a Consensus process among expert healthcare professionals who are involved in the IBD management. The first step was to make a systematic review of the literature to identify the main domains of SoC in IBD. The panel identified three main domains: structure, processes, and outcomes (117). These results served as a basis to set up a list of statements which should identify the essential and desirable characteristics of the structure of an IBD unit, processes for diagnosis, treatment, and follow-up of IBD patients, and outcome to measure the quality of care. The final list of statements was voted during a Consensus meeting held in Copenhagen in 2019 by a multidisciplinary panel of experts and by the ECCO National Representatives, and published as an ECCO position paper (118). Because of the wide heterogeneity across countries and local situations, this position paper was intended to be a starting point for further definition of SoC in IBD that could be adopted, adapted or revised at national and local level. The ECCO is now planning a further step to harmonize SoC in Europe through the E-Quality project, which should identify gaps between the desired SoC and local reality in order to improve knowledge and remove barriers for a high-quality service for IBD patients (119).

## Predict

### Predicting relapse through faecal markers in IBD

IBD is characterized by dysregulated activity of gut-associated lymphoid tissue, leading to overly active intestinal inflammation. This, in turn, leads to chronic inflammatory symptoms, including diarrhoea and abdominal pain, and possible progression toward bowel wall fibrosis and penetrating complications, i.e., fistula and abscesses (120).

Similarly, to other autoimmune conditions, despite maintenance treatment, IBD follow a relapsing–remitting course, as inflammatory flares are interspersed with periods of symptomatic remission. The possibility to predict in advance disease exacerbations would greatly help in the management of IBD patients, as anti-inflammatory therapy escalation before symptomatic flares could prevent their occurrence and potentially positively impact the course of the disease (120). Traditionally, endoscopy has been considered the technique of choice to achieve this goal, as endoscopic activity has been associated with both short and long-term clinical outcomes in UC and CD patients (121–123). Endoscopy, however, has several limitations, including costs, availability, and acceptability by patients, which prevent a more widespread use of this technique for the purpose of relapse prediction. Hence, several efforts have been made over the last few years to discover and validate biomarkers able to predict inflammatory flares in asymptomatic patients, both in UC and CD patients. In the latter clinical condition, a significant proportion of studies focused on the identification of biomarkers predicting post-operative endoscopic and clinical relapse, in order to replace the predominant role of endoscopy in this clinical setting. Even if initial studies suggested a potential role for circulating inflammatory markers, including C reactive protein (CRP) levels, in predicting relapse in asymptomatic patients with CD (124), the current focus has been put on faecal biomarkers, owning to their high specificity and reduced invasiveness.

Faecal biomarkers used in IBD are direct products of inflammatory cells which infiltrate the intestinal mucosa during disease activity. Calprotectin, a protein which constitute 60% of the cytosolic protein in human neutrophils, is a calcium binding heterocomplex of S100A8 and S100A9, whose stool levels are proportional to the influx of neutrophils into the gut lumen, and therefore closely reflects mucosal inflammation (125). Other neutrophil-derived proteins, i.e., faecal lactoferrin and S100A12, are currently being studied as a potential alternative to faecal calprotectin in IBD patients, even if additional studies will be needed to determine their comparative values. Nevertheless, a large number of studies have been carried out demonstrating that levels of these biomarkers, and particularly those of faecal calprotectin, significantly correlate with the endoscopic and histological activity in both UC and CD patients (126).

More than 30 studies have so far evaluated the role of faecal calprotectin in predicting clinical relapse in patients with quiescent IBD (127–136). These studies widely differ in terms of population enrolled, faecal calprotectin cut-off value and method used to assess its levels, follow-up duration and definition of relapse. These limitations explain the extreme heterogeneity of results obtained, as sensitivity and specificity values for predicting relapse range from 28 to 100% and 43 to 52% in CD, and from 31 to 100% and 63 to 100% in UC patients. By considering data from six studies, a meta-analysis by Mao et al. calculated a pooled sensitivity and specificity of faecal calprotectin in predicting relapse in quiescent IBD to be 78 and 73%, respectively, in 672 adult IBD patients (318 UC and 354 CD) (137). Similar figures were observed regarding the role of faecal calprotectin in predicting post-operative recurrence. In the POCER study, applying a faecal calprotectin threshold of 100 μg/g, sensitivity and specificity of this test for detecting a meaningful endoscopic relapse (i.e., endoscopic Rutgeerts’ score ≥ 2) was 89 and 58%, respectively. Authors of the study calculated that applying an algorithm based upon calprotectin values at 6 months following intervention could allow 41% of patients to avoid colonoscopy (138). In a meta-analysis performed by Tham et al., when a faecal calprotectin threshold of 150 μg/g was used, a pooled sensitivity and specificity for of 70 and 69%, respectively, was calculated in the post-operative setting (139). Indeed, the calprotectin cut-off to be used must consider several variables. First, there are differences between CD and UC. For CD, a cut-off of 250 μg/g discriminates disease activity and predicts disease flare (140). For UC, different cut-offs are able to discriminate remission (<150 μg/g), mild (150–200 μg/g), or moderate/severe (>200 μg/g) disease. Finally, according to a recent expert opinion, a cut-off of <40 μg/g indicates deep IBD remission (140).

### Predicting response and toxicity to thiopurines in IBD

Thiopurines, azathioprine, 6-mercaptopurine and thioguanine, have long been used for the medical treatment of IBD (141–143). They have been shown to be effective in maintaining remission in patients with IBD, effectively helping to reduce the rate of surgery in both CD and UC patients, the post-operative relapse rate in CD and the risk of colorectal cancer associated with IBD. Furthermore, when used in combined therapy, they improve the pharmacokinetics of anti-TNF monoclonal antibodies (144). However, with the continuous advent of new biological therapies with different mechanisms of action, the role of thiopurines is being questioned (145).

Up to 20% of patients will respond inadequately or develop toxicity to thiopurine (146), requiring treatment interruption or modification. The availability of predictors for thiopurine response and toxicity allows us to customize this treatment for patients with IBD.

The ability to predict who will respond to thiopurine therapy and to maximize the likelihood of responding earlier in the course of the disease would allow clinicians to tailor therapy earlier, with the goal of altering the natural history of the disease.

Obviously, pre-treatment predictors would be the ideal way to personalize thiopurine therapy in IBD patients.

Several clinical predictors [intestinal site, body mass index, disease activity, disease duration, early or late introduction of thiopurines, ethnicity, sex, etc.] were explored with conflicting results (147). Thiopurine metabolites, thiopurine-S-methyltransferase [TPMT] activity, TPMT polymorphisms and deletion of GSTM1, a polymorphism of the gene encoding glutathione-S-transferase, the enzyme responsible for converting azathioprine in 6-mercaptopurine, they have been studied as predictors of response to thiopurine (147). The use of thiopurine is undoubtedly hindered by the high incidence of adverse drug reactions affecting up to 25% of people who take them, leading to discontinuation of the drug in 17% of patients (148). Side effects often occur in the first few months. Consequently, the ability to predict which patients might develop these potentially serious side effects would be of great use in clinical practice. Toxicities addressed will include myelotoxicity, hepatotoxicity, pancreatitis, alopecia, gastrointestinal and flu-like symptoms and complications associated with Epstein–Barr virus. Thus, while monitoring of thiopurine metabolites shows utility in those already initiated with thiopurine, pharmacogenetic tests, which already play a significant role in preventing toxicity, show some promise in predicting response.

### Predicting response to biologics in IBD

Since the introduction of TNF-alpha inhibitors in the IBD therapeutic armamentarium, other monoclonal antibodies targeting different inflammatory mechanisms have been developed. Namely, vedolizumab targeting alpha4-beta7 integrin involved in T cell intestinal homing and ustekinumab blocking p40, a subunit shared by both by interleukin (IL) 12 and 23 are now available for patients.

Despite the increasing number of therapies available, a high rate of primary failure is observed. Overall, about 30–40% of patients do not respond to TNF inhibitors and those in which an initial clinical benefit is reached tend to lose response and experience clinical relapse over time (149, 150). Similar results have been observed with vedolizumab (151, 152) and ustekinumab (153, 154). In this situation patients are at risk of multiple therapeutic failures with an increased risk to develop complications and permanent organ damage due to uncontrolled inflammation. For these reasons, the identification of predictive factors describing the most appropriate patient for a specific mode of action is currently concentrating large part of research efforts in the IBD field.

Moving from the concept “one drug fits all” to a more personalized approach to patients, different putative predictive factors have been considered that can be divided in different subgroups: patient-related factors, disease-related factors, biomarkers, immune-system-related factors, and microbiota composition. Most of the association studies aiming at identifying predictive factors have been conducted in patients treated with TNF inhibitors and only few have been explored to predict response to vedolizumab and ustekinumab.

Age, gender, and body mass index as predictive factors of response to infliximab reported contrasting results with some studies reporting an association while other failed to do so (reviewed in (155)). Though well defined as risk factor for disabling disease in Crohn’s disease (CD), the role of smoking in predicting response to anti-TNF agents is uncertain (156, 157).

As for disease characteristics, post-hoc analysis from anti-TNF agents registrative trials have found longer disease duration, fibro-stenosing behavior and ileal localization to be associated to worse outcome in anti-TNF-treated CD patients. Overall, the effect of these factors, although not systematically confirmed, might be reconducted to the progressive development of fibrotic ileal stenosis in long-standing CD, a condition that is unlikely to respond to any biologic therapy. Accordingly, the predictive role of these factors in UC is less defined (155).

Finally, biomarkers of inflammation such as CRP and faecal calprotectin have been associated in some studies to higher response rate to anti-TNF agents in CD (158–162) and UC (163–165). Whether more severe inflammation is required for anti-TNF agents to better exert their effect or CRP and faecal calprotectin merely support the presence of intestinal inflammation in patients otherwise affected by symptoms caused by other conditions (e.g., IBS) remains unclear.

While GWAS identified several gene variants associated with the increased risk to develop IBD and some of them were shown to be associated with specific disease phenotype and disease course severity, few have been linked to response to anti-TNF agents. Variants of genes involved in cell apoptosis have been associated to infliximab response while the low affinity immunoglobulin gamma Fc region receptor III-A (FCGR3A) and HLA-DQA1*05 were shown to be associated to antidrug antibody formation and secondary loss of response in CD (166, 167).

A few studies have also assessed the response to therapies in relation to anti-drug antibodies and trough levels, with conflicting results (168). In a previous study, the presence of anti-drug antibodies and low trough levels was predictive of early failure with biosimilar infliximab (169). In another, retrospective study, the baseline infliximab trough levels were not predictive of IBD relapse over the following 2 years (170). Hence, therapeutic drug monitoring for this purpose is uncertain.

In addition to the limited results obtained to identify patient predisposition to respond to biologics, genetic analysis does not consider the dynamic nature of the inflammatory process characterizing IBD. To identify factors that may influence response to therapy in a specific phase of IBD course, analysis of gene expression at mRNA and protein level in the peripheral blood and the intestinal mucosa have been performed. For instance, low expression levels of the triggering receptor expressed on myeloid cells (TREM-1) in the peripheral blood of IBD patients was predictive of primary non-response to anti-TNF therapy (171). However, in another independent CD cohort, downregulation of TREM-1 was predictive of mucosal healing (172). Similarly, serum and tissue expression of oncostatin M was also associated with primary non-response to infliximab and golimumab (173, 174). More recently the expression of membrane -bound TNF and alpha4-beta7 at mucosal level detected by fluorescent-adalimumab and vedolizumab in conjunction with confocal endomicroscopy was shown to predict response in small cohorts of patients treated with adalimumab and vedolizumab, respectively, (175, 176). Additionally, in a very recent study, prediction of response to biologics was assessed through a computerized image analysis of probe confocal laser endomicroscopy (pCLE) *in vivo* and by assessing the binding of fluorescent-labeled biologics *ex vivo* (177). The authors found an 85% and an 80% accuracy in predicting response to therapy in UC and CD, respectively. Additionally, a good prediction to response to anti-TNFs was noticed through a panel of expressed genes (AUC > 0.7).

Microbiota composition has been also explored as predictive factor of response to biologic therapy. Metagenomic analysis of stool samples from patients commencing vedolizumab therapy showed higher alpha-diversity and greater abundance of Roseburia inulinivorans and a Burkholderiales species in CD who achieved remission at week 14 (178). Interestingly, in this study the model incorporating metagenomic and clinical data performed better than the model considering metagenomic data alone.

Most of the aforementioned studies exploring potential predictive factors are biased by the limited number of patients analyzed and in most of the cases by the lack of a validation in a separate independent cohort of patients. The future will see the integration of datasets from different “omic” approaches, as in the case of the Endo-omic study (177), in addition to clinical characteristics in the attempt to define a reliable patient profile to predict response to biologics.

## Prevent

### Preventing flare-up and dysplasia through advanced endoscopy in IBD

Endoscopy is the gold standard for diagnosis, surveillance and assessment of luminal disease activity and extent in IBD patients (115). In recent years, several advanced endoscopic imaging and artificial intelligence techniques were introduced, allowing a detailed analysis of the endoscopic images for precise and reproducible quantification of early inflammatory and dysplastic changes at a microscopic and ultrastructural level. Currently, the main goal of advanced endoscopy in IBD is the assessment of deep mucosal healing looking beyond the standard definition of endoscopic remission for early prevention of disease flare-up and tight drug monitoring (179, 180).

In 2020, the Picasso study, a large, international, multicenter, prospective trial, demonstrated that virtual chromoendoscopy can predict valuable clinical outcomes with the similar accuracy provided by histology in UC patients (181, 182). The same study group is now developing two different operator-independent AI systems to detect deep endoscopic remission on virtual chromoendoscopy video-images (183) and histologic remission on digitalized histological slides (184) to predict the risk of a flare-up at 12 months, hereby enabling clinicians of novel diagnostic tools and strategies for precise patient-tailored management.

In 2021, Bossuyt et al. have described two different endoscopic prototypes for computer-aided assessment of mucosal inflammation in UC introducing the firsts operator-independent scoring systems in the field of IBD. Both prototypes can be activated in real time, do not require contrast agents and are supported by computer-aided algorithms (machine learning algorithm) that as opposed to other types of artificial intelligence (likewise convolutional neural network) must be fed structured data and are not able to cope with unforeseen circumstances. The red density score (185) provides a quantitative assessment of disease activity based on the redness map and vascular pattern recognition, which correlates well with both endoscopic and histopathological disease activity. The second artificial intelligence-powered endoscope provides a single short wave-length monochromatic LED light illumination enabling a magnified visualization of inflammatory changes on the pericryptal vessels (186). This artificial intelligence system predicts histologic remission with higher accuracy (86%) as compared with standard endoscopic scoring systems (74% with Mayo endoscopic sub score, 79% with UC endoscopic index of severity). Larger studies and validation in independent cohorts are on the way to confirm the preliminary results obtained with these colonoscopy prototypes.

In 2022, Maeda et al. have described a novel artificial intelligence system working on endocytoscopy images able to predict clinical relapse during 12 months of follow-up in UC patients in clinical remission from a single referral Japanese center. The endocytoscope was integrated into the distal tip of a standard colonoscope to collect ultra-magnified images (×520) in real time. Notably, irrespective of the operator expertise, this AI system predicts clinical relapse better than expert assessment of endoscopic remission (Mayo endoscopic score ≤ 1) and comparably than expert assessment of endoscopic deep remission (Mayo endoscopic score = 0) or histologic remission. Other studies either based on recorded colonoscopy videos (187–189) or prospectively collected fixed images (190) have produced confirmatory evidence on the promising role of AI in refining mucosa activity assessment (187–189) to predict prognosis in UC patients (190).

The diagnosis of colorectal dysplastic changes in patients with longstanding IBD colitis or primary sclerosing cholangitis is still an unmet issue (191). Small but clinically relevant non pedunculated colorectal lesions are often overlooked during surveillance colonoscopy due to their subtle or inflammatory-like appearance (192). In addition, the optical characterization of neoplasia is still challenging in IBD colitis, even using advanced endoscopic technology in expert hands (193). Within this context, the future goal of artificial intelligence is the detection and characterization of colitis-associated dysplasia at an early stage, revealing non advanced colorectal lesions when still suitable for curative endoscopic resection or for timely prophylactic surgery based on a patient-tailored multidisciplinary approach (194). The first artificial intelligence-assisted detection of colitis-associated neoplasia has been recently video-documented (195). However, the artificial intelligence systems successfully adopted for sporadic colorectal adenoma detection and characterization were not trained in active and post-inflammatory changes, and therefore need further developing in the setting of IBD.

### Preventing infections in IBD

The treatment of IBD has been revolutionized in recent years by the increasing use of immunosuppressants, biologics and small molecules. Although these powerful drugs have proved effective in controlling disease activity, they can lead to a condition of immunosuppression, consequently increasing the risk of new infections or reactivation of latent infections (196). In this context, the evaluation of the immunological and serological status of patients with IBD is extremely useful in proposing adequate preventive measures against such infections, first of all vaccines (197).

In this sense, the guidelines of the Infectious Diseases Society of America distinguish patients with low- and high-level immunosuppression (198). This distinction is crucial in evaluating the advisability of immunizing patients using live vaccines.

According to various evidences, the main risk factors for opportunistic infections are advanced age (OR 3.0 for patients>50 years) (199) and the simultaneous use of multiple immunosuppressive drugs, since the transition from a single immunomodulator to combination therapy increases the relative risk of infections from 2.9 to 14.5 (200) Considering TNF-α inhibitors alone – which are in themselves sufficient to define high-level immunosuppression, as previously mentioned – the TREAT registry showed an increased risk of serious infections with these drugs (HR 1.47) (201). Regarding the risk of infections with new biological therapies approved in IBD, vedolizumab – a monoclonal gut-selective antibody that targets integrin α4β7 and ustekinumab a monoclonal antibody directed against the p40 subunit of IL-12 and IL-23 do not appear to be associated with important infectious complications (202, 203). Tofacitinib – an orally administered non-selective JAK inhibitor – is associated with an increased risk of herpes zoster infection in patients with IBD, with an observed incidence of 4 herpes zoster infections per 100 patient-years (204). In this regard, the availability of Shingrix^®^, a new recombinant non-live vaccine already approved for adults aged 50 and over against virus reactivation, could be of great help in patients who will be treated with Tofacitinib and future drugs of the same class (205).

All patients with IBD must be tested for the hepatitis C and B virus (HBsAg, anti-Hbs, anti-HBc) to assess the infectious status (current, past, inactive carrier) or vaccination status. In patients with evidence of active HBV infection, HBeAg, anti-HBe and HBV DNA must also be evaluated (206). Obviously, the HBV vaccine is recommended in all seronegative patients.

In patients who will be treated with TNF-α inhibitors, a careful preliminary assessment of the presence of latent tuberculosis is mandatory, since there is a high risk of reactivation of the infection with these drugs (207). If treatment with thiopurines is planned, screening should be performed to ascertain the serological status against EBV (208, 209).

Studies conducted in women with IBD demonstrated a high frequency of Human Papilloma Virus (HPV) associated Pap-smears abnormalities (210). In this setting, immunosuppressive drugs could increase the risk of persistent HPV infection and ultimately cervical cancer.

Infections caused by Neisseria meningitidis can evolve into a disease with high mortality, if not recognized and promptly treated. Meningococcal infections are endemic in Western countries, and the annual incidence of invasive meningococcal disease varies in multiyear cycles (211, 212).

The ongoing pandemic of COVID-19 caused by infection with SARS-CoV-2 is worthy of a brief comment in the context of vaccinations for patients with IBD. COVID-19 has raised substantial concerns for patients with IBD who are receiving immunosuppressive agents. Patients with IBD do not appear to be at greater risk of infection by SARS-CoV-2 compared to the general population, and current treatments are not associated with worse prognosis. However, clinicians should be cautious about the use of systemic steroids for treatment of COVID-19 (213).

Recently, a consensus on be-half of the International Organization for the Study of Inflammatory Bowel Disease provided several statements supporting SARS-CoV-2 vaccination in patients with IBD regardless the immune-modifying therapies (214). Generally, all IBD patients should be vaccinated for the following diseases: tetanus, diphtheria and polio, measles, mumps and rubella, HBV, influenza, varicella, HPV, pneumococcus, and meningococcus (215).

Physicians, and in particular gastroenterologists, should be encouraged to conduct a structured interview of all IBD patients at diagnosis, about the immunization status and the definition of a vaccination plan before the start of immunosuppressive treatment (215).

### Preventing post-surgical recurrence in IBD

Only 10 years ago, the idea that a surgeon should be involved in the prevention of post-surgical recurrence of IBD would have been at least considered imaginative, but nowadays surgery has great an impact on the patients’ clinical history, including the disease recurrence.

In UC surgery is curative, even if in very selected cases, a segmental colectomy could be considered to avoid total proctocolectomy (216). However, the patient could experience post-surgical consequences such as incisional hernias, stenosing or fistulizing complications of the ileo-pouch-anal anastomosis, and adhesions potentially causing obstructions and reduced fertility in women. The standardization of restorative proctocolectomy procedure, taking into account all the problems emerged during the past years of surgical practice, and the new technologies available, have significantly reduced all the major drawbacks, in particular, the long-term pouch disfunction and failure, giving the patients the possibility to avoid definite ileostomy (217–219). The extended use of laparoscopy in referral centers has not only drastically reduced perioperative complications, length of hospitalization, and incisional hernias, but it left the women’s fertility preserved through a notable reduction in adhesions (219–222). It is worth pointing out that laparoscopy can be used to complete a restorative proctocolectomy even in case of staged procedures, with very low conversion rate (220).

Looking at the other side of the IBD-moon, in the “traditional” view of CD clinical history, surgeons were only involved when any pharmacological option had been failed and perforating, or stenotic complications had been occurred. Due to this gastroenterologists’ “last-resort” approach, the surgeons, for their part, have tried to focus on identifying all the risk factors for post-operative complications that they could have acted upon. Thanks to the evidence from surgical trials, performed at the turn of the 21st century, today we know that steroids and anti-TNF agents should be withdrawn before surgery, nutritional status checked and amended, deep venous thrombosis prevented, intra-abdominal abscesses radiologically drained, and acute obstruction managed conservatively, exploiting a “postpone and optimize” strategy (223). Furthermore, some risk factor for post-operative recurrence has been identified such as a genetic predisposition, a paediatric diagnosis, the need for surgery within 1 year of diagnosis, a smoking habit, a penetrating disease behavior in the terminal ileum and colon, a stricturing behavior associated with upper gastrointestinal location, the presence of perianal disease, and the bowel wall thickening and the endoscopic recurrence within 1 year after surgery (224–228). However, focusing on anastomotic post-surgical recurrence using the score proposed by Rutgeerts and colleagues, it became evident that different anastomotic configurations presented different long-term recurrence, with side-to-side anastomosis (either isoperistaltic or anisoperistaltic – also known as functional end-to-end) having the better results over all the other anastomotic configurations (end-to-side, end-to-end, and side-to-side with blind loops) (223, 229). Recently, a particular kind of functional end-to-end anastomosis, proposed by Kono et al., has been claimed to drastically reduce both post-operative complications and long term endoscopic and surgical recurrence, but a direct comparison with a stapled similar anastomosis, considering also postoperative adjuvant treatment, is still lacking (230, 231). Another argument of actual debate is the treatment of the mesentery during ileo-colic retention, since a group from Limerick supported a role of mesentery removal in preventing post-surgical recurrence. On the one hand, the well-known low recurrence rate observed on the site of a strictureplasty, a technique where both the diseased bowel and mesentery are preserved, is in stark contrast with this hypothesis (222, 224). On the other hand, a large retrospective study and a retrospective comparative analysis, both performed on prospective databases, failed to find any evidence and strengthened the importance of post-operative prophylaxis with biological treatment (231–234).

## Unmet needs in IBD

Despite the recent developments in the diagnosis and monitoring of IBD (235–237), together with the new biologics and small molecules which expanded our therapeutic armamentarium for treating these diseases (238–240), several patients still suffer from disabling bowel symptoms and relevant disease complications (241, 242). Moreover, patients with IBD experience impairment of health-related quality of life, fatigue, depression, and anxiety (243, 244). Thus, many questions remain to improve the management of IBD.

Both CD and UC are heterogeneous diseases, with significant differences in terms of disease location, activity, onset and course in each patient. However, current treatment strategies suggest a standard approach to all patients, because of the limited evidence regarding the existence of useful and easy-to-use predictors of response to therapy (245). In the last decade, with the novel drugs available, various efforts have been made to fill this gap, by evaluating the individual and molecular specificities of the disease, identifying new biomarkers and stratifying individual patients’ risk factors in its early stages (246). Nevertheless, the incorporation of these predictors in routine clinical practice is not yet established.

IBDs are progressive disorders in which chronic inflammation may lead to disease progression, disability and complications (247–250). Various studies have shown that early use of biologic drugs is associated with improved clinical outcomes and, therefore, the accurate identification of patients who may benefit of early intervention seems to be crucial. Although several studies identified and suggested biochemical markers (e.g., faecal calprotectin, C-reactive protein, oncostatin M), clinical indices (i.e., early disease onset, smoking, use of steroids, extended disease, perianal involvement), genetic tests, proteomic, metabolomic or metagenomic and radiomics or imaging-based biomarkers, to identify higher-risk patients, prospective outcome data corroborating these variables are lacking. Thus, more objective and prospectively validated biomarkers are needed in order to implement a precision-medicine approach in IBD (246).

To date, registration trials demonstrated that current available treatments for both UC and CD do not achieve remission rates over 50%, underlining a therapeutic ceiling in the management of both diseases and potential challenges that need to be addressed (251). For this reason, a treat-to-target approach with tight monitoring of the disease during maintenance treatment has been investigated, emphasizing the potential to improve drug efficacy, but also highlighting the fact that patients still may present with complications in at least 30% of the cases in the mid-term (252). Moreover, treatment goals are challenging to achieve in clinical practice since they depend on many variables, including the disease duration, timing of treatment initiation, drugs available, compliance to treatment, and duration of therapy. Therefore, new and more effective therapies for IBD are needed. On the other hand, other questions arise from the use of current available treatments: which drug should be used as first-line treatment, which drug should be used after a failure, when to start or stop a drug, how to best adjust timing and dosing (i.e., the role of therapeutic drug monitoring). Moreover, should we combine biologics and small molecules to improve the efficacy without taking into consideration safety issues? Unfortunately, to date most of the comparative efficacy data can be retrieved from systematic reviews and network meta-analyses because head-to-head trials are in their early development (253, 254), whereas evidence regarding the combined use of drugs with different mechanisms of action is very limited and the risk-safety and cost–benefit analyses are still unsatisfactory (255).

There are other different clinical scenarios where there is a lack of evidence regarding the correct management of IBD. For instance, for the treatment of perianal fistulizing CD, infliximab represents the best-studied drug showing a long-term healing rate lower than 50%, while vedolizumab and ustekinumab have been only evaluated in retrospective studies and post-hoc analyses (256). Limited evidence is also available for the use of anti-JAK, mesenchymal stem cells, and combination of biological therapies in this setting. Likewise, the treatment of acute severe UC suffers from the same limitations mentioned above for perianal fistulizing CD, with a few treatment modalities assessed to date (257). Ulcerative proctitis (UP) represents another example of difficult-to-treat disease, since approximately half of these patients are refractory to conventional medical treatment and the efficacy data from registration biologic trials, where UP patients are systematically excluded, is limited (258). Prevention of post-operative recurrence is still a controversial field, although a recent systematic review and meta-analysis showed that anti-TNF therapies, alone or in combination, seem to be the best way for preventing endoscopic recurrence (259). However, which patients should be treated, the best timing to initiate biologic therapy in the post-operative setting, as well as the role of new biologic therapies, are all features that need to be investigated. Further, there is an urgent need to identify new therapeutic targets and new effective molecules for tackling intestinal fibrosis in CD. The uncertainties regarding the potential detrimental effects of such drugs for CD (i.e., perforation, worsening of intestinal inflammation) (3). In fact, there are no available therapies for this purpose, and, for this reason, the rate of surgery in CD is still high.

Also the frail patient, usually having an age over 65 years, with various comorbidities including the cardiovascular ones, represents a challenging clinical situation, since the high risk of developing significant adverse events (i.e., infections) leads frequently to adopt sub-optimal IBD therapy, as shown by chronic corticosteroid use and elevated surgical rates (260). In this type of patient, the use of gut-selective immunosuppressants seems to have the potential to reduce the risk of adverse events, but further studies are needed. Finally, other settings requiring further data and advancements are relative to the use of novel non-invasive markers for monitoring the disease, with a better correlation with mucosal inflammation and disease progression, and the improved control of fatigue together with anxiety and depression which are able to affect greatly the disease course and therefore should be considered in its management (261).

## Conclusions and outlook

Due to its multifactorial pathogenesis, its clinical manifestations, and its long-life clinical impact, IBD represents a typical example of a complex disease (7), making its clinical management challenging for physicians. As we have discussed, age- and gender-specific issues, specific comorbidities, availability of different biologics and small molecules with different route of administrations, and socioeconomic and cultural factors, are just some of the variables that affect decision-making in IBD. Additionally, despite the availability of novel drugs, clinical and endoscopic remission rates on a long-term are still poor and proper drug positioning is uncertain. Hence, an integrated effort is required to meet the key objectives needed to pursue targeted treatment. The 4P framework seems to be the best currently available tool for clinicians that could be applied for tailoring interventions in day-by-day clinical practice in the IBD field. Table 2 summarises the main variables to be considered in the 4P medicine framework that could prove useful in the overall IBD management, along with a possible future trend per each area.

**Table 2:** Main variables to be considered in the 4P medicine framework that could prove useful in the overall management of inflammatory bowel disease (IBD).

4P sub-area	Variables to be considered	Future trends
Personalize	Age groups (i.e., childhood, adulthood, elderly) Sex and gender (i.e., male, female, cisgender, transgender, others) Co- and multi-morbidity Specific patient populations (i.e., pregnancy, oncological, immunosuppressed) Socioeconomic status in relation to coping abilities, disease understanding, impact on work-life balance Adherence to medications Patients’ preferences	Research will focus on groups that are usually underrepresented in both clinical trials and observational studies Both sex and gender will be included and separately analyzed in clinical trials Real-world, controlled studies are warranted to study the safety and efficacy of the available drugs for treating IBD Patient-reported outcome always included in prospective studies
Participate	Communication with IBD patients Facing physical, psychological, and sexual disability Resilience and stigmatization Patient associations and public social events	Physician will be trained on how to communicate to IBD patients, as well as on how to assess disability, resilience, and stigmatization Patients will be more involved in public social events for raising awareness on IBD
Predict	Prediction of likelihood of developing IBD Prediction of likelihood of developing IBD complications Prediction of response to IBD treatment	Artificial intelligence and machine learning will allow early prediction of developing IBD and its complication and will allow a personalized approach to therapies More serum and faecal markers will be made available
Prevent	Prevention of disease flare or recurrence and dysplastic/neoplastic complications Prevention of opportunistic and vaccine-preventable infections Prevention of infertility/sexual issues, disability, and psychiatric illness deriving from IBD	Advanced endoscopic techniques will be made available for early detection of dysplasia and colorectal cancer Safe and new vaccine, also including the novel mRNA-based vaccines, will be made available and specifically tested in the IBD population Prevention of infertility and sexual issues, or of any other form of disability and psychological distress, will be key in the overall management of IBD patients

In the future, new tools for predicting important disease outcomes may become available and are eagerly awaited. Particularly, classical statistics, such as linear regression or advanced regression analyses, may fail to capture a meaningful sense of all the variables called into question in IBD. In fact, the increase in data complexity makes classical statistical inference less tractable (262). Recent advancements in the research field of machine learning have fostered the development and deployment of healthcare-focused algorithms. In this context, historical patient data are used to draw correlations and build machine learning models able to predict, with a known confidence, what is likely to happen to a patient, given their current medical condition. The use of artificial intelligence algorithms (263) may be able to capture and decipher complex, nonlinear relationships, that otherwise would be left unconsidered, and could be consider as an upgrade of the 4P medicine. So far, the only application of artificial intelligence in the field of IBD is that of endoscopy, field in which it proved useful in detecting preneoplastic alterations (264). A broader clinical application of artificial intelligence should be sought in future studies.

## Author contributions

MVL and ADS introduction, conclusions, and outlook. MLS tailoring treatment of IBD in pregnant women. LB tailoring treatment of IBD in cancer patients. RC tailoring treatment of IBD in patients with superimposed viral infections. DP how to communicate with IBD patients. LP disability in IBD. MVL stigmatization and resilience in IBD. GF quality of care standards in IBD. FC predicting relapse through faecal markers in IBD. SA predicting response and toxicity to thiopurines in IBD. MF predicting response to biologics in IBD. GT preventing flare-up and dysplasia through advanced endoscopy in IBD. AO preventing infections in IBD. GMS preventing post-surgical recurrence in IBD. ES unmet needs in IBD. GC, MVL, ADS, RD’I, MD, MV, GM, AK, and GCS overview and revision for important intellectual contents. All authors contributed to the article and approved the submitted version.

## Funding

The publication costs are supported by the network “Rete Aging” – Fondazione IRCCS Policlinico San Matteo.

## Conflict of interest

ES has served as speaker for Abbvie, AGPharma, Alfasigma, EG Stada Group, Fresenius Kabi, Grifols, Janssen, Innovamedica, Malesci, Pfizer, Reckitt Benckiser, Sandoz, SILA, Sofar, Takeda, Unifarco; has served as consultant for Alfasigma, Amgen, Biogen, Bristol-Myers Squibb, Celltrion, Diadema Farmaceutici, Falk, Fresenius Kabi, Janssen, Merck & Co, Reckitt Benckiser, Regeneron, Sanofi, Shire, SILA, Sofar, Synformulas GmbH, Takeda, Unifarco; he received research support from Reckitt Benckiser, SILA, Sofar, Unifarco. DP has received speaker’s fee from Takeda, Pfizer, MSD, Janssen, MSD, and a grant from Amgen and Pfizer.

The remaining authors declare that the research was conducted in the absence of any commercial or financial relationships that could be construed as a potential conflict of interest.

## Publisher’s note

All claims expressed in this article are solely those of the authors and do not necessarily represent those of their affiliated organizations, or those of the publisher, the editors and the reviewers. Any product that may be evaluated in this article, or claim that may be made by its manufacturer, is not guaranteed or endorsed by the publisher.
